# Serious adverse neonatal outcomes such as 5-minute Apgar score of zero and seizures or severe neurologic dysfunction are increased in planned home births after cesarean delivery

**DOI:** 10.1371/journal.pone.0173952

**Published:** 2017-03-20

**Authors:** Amos Grünebaum, Laurence B. McCullough, Birgit Arabin, Frank A. Chervenak

**Affiliations:** 1 Department of Obstetrics and Gynecology, Weill Cornell Medicine, New York, New York, United States of America; 2 Center for Mother and Child, Philipps University, Marburg, Germany; 3 Clara Angela Foundation, Berlin, Germany; University of Washington, UNITED STATES

## Abstract

The United States is with 37,451 home births in 2014 the country with the largest absolute number of home births among all developed countries. The purpose of this study was to examine the occurrence and risks of a 5-minute Apgar score of zero and neonatal seizures or serious neurologic dysfunction in women with a history of prior cesarean delivery for planned home vaginal birth after cesarean (VBAC), compared to hospital VBAC and hospital birth cesarean deliveries for term normal weight infants in the United States from 2007–2014. We report in this study outcomes of women who had one or more prior cesarean deliveries and included women who had a successful vaginal birth after a trial of labor after cesarean (TOLAC) at home and in the hospital, and a repeat cesarean delivery in the hospital. We excluded preterm births (<37 weeks) and infants weighing under 2500 g. Hospital VBACS were the reference. Women with a planned home birth VBAC had an approximately 10-fold and higher increase in adverse neonatal outcomes when compared to hospital VBACS and hospital repeat cesarean deliveries, a significantly higher incidence and risk of a 5-minute Apgar score of 0 of 1 in 890 (11.24/10,000, relative risk 9.04, 95% confidence interval 4–20.39, p<.0001) and an incidence of neonatal seizures or severe neurologic dysfunction of 1 in 814 (Incidence: 12.27/10,000, relative risk 11.19, 95% confidence interval 5.13–24.29, p<.0001). Because of the significantly increased neonatal risks, obstetric providers should therefore not offer or perform planned home TOLACs and for those desiring a VBAC should strongly recommend a planned TOLAC in the appropriate hospital setting. We emphasize that this stance should be accompanied by effective efforts to make TOLAC available in the appropriate hospital setting.

## Background and objectives

Out-of-hospital (OOH) births in the United States (US) are births occurring outside the hospital and include home and birth center births. OOH births increased from 2009 to 2014 by 80.2% from 32,596 to 58,743 (0.79%-1.47% of all live births).[1] Home births (HB), which are part of OOH births increased by 77.3% [1] from 2009–2014 thus making the US with 37,451 home births in 2014 the country with the largest absolute number of home births among all developed countries, surpassing the approximate 28,000 home births per year in the Netherlands, where home births have decreased over the last decades, though the proportion of home births in the Netherlands is still higher.[2] Despite the American College of Obstetricians and Gynecologists' (ACOG) statement that because of it’s increased risk a prior cesarean delivery is a contraindication for a home birth,[3] about 1 in 23 home births in the US are women with prior cesarean delivery.[4]

The purpose of this study was to examine the occurrence and risks of a 5-minute Apgar score of zero and neonatal seizures or serious neurologic dysfunction in all women with a history of prior cesarean delivery for planned home birth VBAC, hospital VBAC and hospital birth cesarean deliveries for term normal weight infants in the United States from 2007–2014.

## Materials and methods

This is a retrospective cohort study. Data were obtained from the National Center for Health Statistics (NCHS) of the US Centers for Disease Control (CDC) birth certificate data for 2007–2014. The CDC files contain detailed information on each of the approximately 4 million births in the United States each year. Data on patient characteristics include birth setting and method of delivery as well as whether a home birth was intended or not as reported on birth certificates filed each year with the states of the United States and compiled by NCHS. These data are publicly accessible on the internet (http://205.207.175.93/vitalstats/ReportFolders/ReportFolders.aspx), where detailed tables can be created and downloaded for further evaluation.

The data that we report in this study are for the 2007–2014 period of women who had one or more prior cesarean deliveries and included women who had a successful vaginal birth after a trial of labor after cesarean (TOLAC) at home and in the hospital, a planned repeat cesarean delivery in the hospital, as well as a repeat cesarean delivery after a failed trial of labor after cesarean (TOLAC) in the hospital. We excluded preterm births (<37 weeks) and infants weighing under 2500 g. This study therefore includes only term births (deliveries ≥37 weeks) and infants weighing ≥2500 g.

The home birth variable on the Standard Certificate of a Live Birth distinguishes between an intended (planned) and a non-intended (unplanned) home birth and therefore encompasses “carefully planned home births with emergency unplanned home births”.[5] We included only the variables in the birth certificate that indicated planned (intended) home births “carefully planned home births “in this study.

We included outcome data on a 5-minute Apgar scores which are well reported on birth certificates, the clinical and prognostic utility of which is well established.[6,7,8] We also included outcome data on neonatal seizures or serious neurologic dysfunction, the category used by the CDC on birth certificate data. The CDC defines a seizure as “any involuntary repetitive, convulsive movement or behavior.” A serious neurologic dysfunction is defined by the CDC as “severe alteration of alertness such as obtundation, stupor, or coma, i.e., hypoxic-ischemic encephalopathy. Excludes lethargy or hypotonia in the absence of other neurologic findings. Exclude symptoms associated with CNS congenital anomalies.” (http://www.cdc.gov/nchs/data/dvs/facwksBF04.pdf last accessed June 8, 2016) Five-minute Apgar score of zero and data on seizure or serious neurologic dysfunction were calculated for home VBACs (women with prior cesarean deliveries who had a vaginal birth), hospital VBACs, and hospital repeat cesarean deliveries. Hospital VBACs served as the reference group. All statistical analyses were conducted in OpenEpi.[9]

### Statistics

Because non-identifiable data from a publicly available dataset were used, our study was not considered human subjects research and did not require review by the institutional review board of Weill Medical College of Cornell University.

## Results

Table 1 shows patient characteristics of planned home VBACs as compared to hospital births for 2014. As in prior studies of planned home births, patients with a planned home VBAC were significantly more likely to be non-Hispanic white, ≥30 years of age, US born, and self-pay.

**Table 1:** Patient characteristics: Hospital repeat cesarean, hospital VBAC and planned home VBAC for 2014.

Characteristics	Hospital Repeat CS	Hospital VBAC	Home VBAC
	N = 418,004	N = 52,946	N = 1,253
**Maternal Race/Ethnicity**			
Non-Hispanic white	51.3%	52.8%	88.5%
Non-Hispanic Black	16.7%	15.1%	3.1%
Hispanic	25.6%	23.1%	6.4%
**Maternal age (yr)**			
<25	16.2%	15.1%	8.8%
25–29	28.1%	29.3%	29.8%
30–34	32.5%	34.0%	38.4%
35–39	18.7%	17.7%	18.2%
>39	4.5%	4.0%	7.1%
Unmarried	34.7%	30.0%	6.5%
**Payment**			
Medicaid	45.0%	42.1%	10.3%
Private	47.1%	47.7%	18.2%
Self-Pay	3.4%	4.2%	62.9%
**Live birth order**			
2nd	55.7%	45.8%	27.8%
3rd	28.7%	27.5%	26.2%
4th	10.2%	13.7%	16.0%
Over 4th	5.0%	12.5%	29.5%
Mother foreign born	24.6%	26.8%	6.7%
**Birth attendant**			
Physician	99.8%	87.1%	1.6%
CNM/CM	0%	12.1%	22.9%
Other midwife	0%	0.3%	55.7%
Other	0.2%	0.5%	18.6%

Women with a planned home birth VBAC had an approximately 10-fold and higher increase in adverse neonatal outcomes when compared to hospital VBACS and hospital cesarean deliveries. Table 2 shows the 5-minute Apgar score of zero and seizures or neurologic dysfunction for the 3 groups. Planned home VBACs had a significantly higher incidence and risk of a 5-minute Apgar score of 0 of 1 in 890 (11.24/10,000, relative risk 9.04, 95% confidence interval 4–20.39, p<.0001) and an incidence of neonatal seizures or severe neurologic dysfunction of 1 in 814 (Incidence: 12.27/10,000, relative risk 11.19, 95% confidence interval 5.13–24.29, p<.0001) when compared to hospital VBACs. Hospital delivery VBACs were associated with non-significant increase in 5-minute Apgar of 0 and a non-significant decrease in neonatal seizures when compared to hospital repeat cesarean deliveries.

**Table 2:** 5-minute Apgar score of zero and seizures or severe neurologic dysfunction in home birth VBACs versus hospital VBACs and hospital repeat cesarean deliveries 2007–2013.

Cohort	n/total	Reciprocals (per 10,000)	RR [95% CI]	P
**5-Min Apgar = 0**				
Planned Home Birth VBAC	7/6,229	1 in 890 (11.24)	9.04 [4–20.39]	< .0001
Hospital Repeat Cesarean Delivery	241/2,575,044	1 in 10,685 (0.94)	0.75 [0.53–1.08]	.121
Hospital VBAC	34/273,522	1 in 8,045 (1.24)	1	
**Seizures or Neurologic Dysfunction**				
Planned Home Birth VBAC	8/6,510	1 in 814 (12.27)	11.2 [5.14–24.42]	<.0001
Hospital Repeat Cesarean Delivery	342/2,574,128	1 in 7,527 (1.33)	1.21 [0.83–1.76]	.315
Hospital VBAC	30/273,401	1 in 9,113 (1.10)	1	

## Comments

### Principal findings

Our study shows that a planned home vaginal delivery of a woman with a prior cesarean delivery is associated with a significantly and markedly increased neonatal risk of a 5-minute Apgar score of 0, and neonatal seizures or serious neurologic dysfunction when compared to hospital deliveries of women with prior cesarean deliveries, either VBACs or repeat cesarean delivery.

### Clinical implications

According to the American College of Obstetricians and Gynecologists, a low 5-minute Apgar score may be one of the first indications of encephalopathy,[10,11] correlates with neonatal mortality in large populations,[12] and clearly confers an increased relative risk of cerebral palsy, reported to be as high as 20-fold to 100-fold over that of infants with a 5-minute Apgar score of 7–10.[10,13,14,15,16]

A successful trial of labor after prior cesarean delivery (TOLAC) has several potential health advantages for pregnant women. Women who have a successful TOLAC with a VBAC avoid major abdominal surgery, have lower rates of hemorrhage and infection, experience a shorter recovery period, and may avoid potential future maternal consequences of multiple cesarean deliveries such as hysterectomy, bowel or bladder injury, transfusion, infection, and abnormal placentation such as placenta previa and placenta accreta.[17]

Obstetricians and other concerned professionals should understand, identify, and correct the reasons why women with prior cesareans want to deliver at home. Hospitals should create a strong culture of safety with the lowest possible risks. In addition, they should attempt to create an environment committed to fewer unnecessary interventions such as preventing first-time cesarean deliveries, and help women experience a more home-birth-like delivery.[18,19,20,21,22]

The absolute risk for uterine rupture in women undergoing (TOLAC) has been reported to be between 0.5 and 4% or between 1 in 200 to 1 in 25,[23,24] and a trial of labor after prior cesarean delivery in the hospital is associated with a greater perinatal risk than is elective repeat cesarean delivery without labor.[25]

Because of lower maternal risks, ACOG recommends that women should be offered a TOLAC and that it should be undertaken only in facilities capable of providing emergency care.[3] ACOG classifies a prior cesarean delivery as a contraindication for a home birth because of the risks associated with a TOLAC, such as the unpredictability of uterine rupture and other complications, and because there is no access to immediate expert neonatal resuscitation.[3] The majority of neonatal hypoxic ischemic encephalopathy in patients with TOLAC occur after rupture of the uterus [3] which can be diagnosed with electronic fetal monitoring and can be best managed with expeditious access to all required personnel, anesthesia care, and an operating room, none of which are available with home births.

Our study showing that planned home VBAC is associated with a significantly and markedly increased risk of a 5-minute Apgar score of zero and neonatal seizures or serious neurologic dysfunction has important implications for the informed consent process for planned out-of-hospital birth. In the ethics and law of informed consent, obstetricians have the professional responsibility to identify medically reasonable alternatives for the management of pregnancy and their benefits and risks.[26] Though a TOLAC and successful VBAC is preferable for maternal benefits, in the context of reducing avoidable neonatal risk, the data reported here strongly support the recommendation that planned home TOLAC may not be medically reasonable, as it may result in serious avoidable neonatal complications, given the preventable, clinically significant absolute and relative risks of adverse perinatal outcomes.

Obstetric providers should therefore not offer or perform planned home TOLACs and for those desiring a VBAC should strongly recommend a planned TOLAC in the appropriate hospital setting.[26,27] We emphasize that this stance should be accompanied by effective efforts to make TOLAC available in the appropriate hospital setting.

### Strength and weakness

The major strength of our analysis is the large sample size for both hospital and home birth over an 8-year period from the most comprehensive and reliable dataset available in the United States.

Our study has several limitations. The quality of data reported in birth certificates can vary,[5,6,7] though most of the data we used is considered to be reliable. Although information on setting, birth attendant, and Apgar scores is reliable in the CDC dataset, data on seizures or serious neurologic dysfunction are less so,[6,7,8] Not all states participate in the birth certificate data, so their applicability to all US states is not proven. For the states reporting, there was a 97.5% compliance rate for indicating presence or absence of seizures or serious neurologic dysfunction. The CDC data on seizures or serious neurologic dysfunction include those of genetic and prenatal origin that might not be related to birth setting. Another limitation is that it is not possible to know from the CDC data whether a 5-minute Apgar score of 0 was effectively a stillbirth that occurred antepartum or intrapartum. We do not believe that this limitation changes our major findings because the vast majority of stillbirths delivered in the hospital are known to be antepartum and not intrapartum.[28,29]

Data on long-term follow-up of neonates would be optimal, but the CDC database does not include such information. An Apgar score of 0 indicates that there are no signs of life (no heartbeat, no breathing or movements). Infants with a 5-minute Apgar score of 0 have a significantly increased risk of mortality and if they survive an increased risk of significant morbidity.[30,31] Survival relates directly to the effectiveness of advanced neonatal resuscitation that is severely limited in home births.

The CDC does not categorize on birth certificates as out-of-hospital births those hospital births that resulted from transfer from out-of-hospital settings where there was an intention for out-of-hospital birth. There is no way to assess from the CDC natality data when intended out-of-hospital TOLAC deliveries are transferred to the hospital, making an intention-to-treat analysis impossible. Unsuccessful planned home TOLACs may be transferred to a hospital and may then become a hospital repeat cesarean with likely more adverse neonatal outcomes. Because these adverse outcomes are attributed to hospital births instead of home births, this would likely make planned home TOLAC even more of a risk than stated.

### Conclusions and implications

Our study results add to and extend the data on the avoidable, greatly increased neonatal risks of home VBAC. [32] These results should become the basis for development of evidence-based guidelines on planned home births for women with prior cesarean delivery. The American Congress of Obstetricians and Gynecologists as well as the Royal College of Obstetricians and Gynecologists have stated that having a prior cesarean delivery is a contraindication for a planned home birth.[17,18] Midwifery organizations in other countries such as the Netherlands, England, and Australia have also recommended against a planned home TOLAC. The American College of Nurse Midwives (ACNM), citing supposed lack of data on outcomes, has not taken an official position on this issue while the Midwives Alliance of North America (MANA), the American home birth midwifery association, supports planned home TOLAC even though studies show that there is an increased risk to the newborn in home births VBACs.[33,34,35,36,37]

As part of the standard practice of the informed consent process, all obstetric providers must disclose the avoidable increased serious neonatal risks of planned home births after cesarean delivery to all women who express an interest in out-of-hospital TOLAC.[23,24] Providing professional guidance with significant, evidence-based information that a planned home birth TOLAC is contraindicated will enhance women’s autonomous decision-making.
